# The Antioxidant Response Induced by *Lonicera caerulaea* Berry Extracts in Animals Bearing Experimental Solid Tumors

**DOI:** 10.3390/molecules13051195

**Published:** 2008-05-27

**Authors:** Maria Iuliana Gruia, Eliza Oprea, Ion Gruia, Valentina Negoita, Ileana Cornelia Farcasanu

**Affiliations:** 1 Institute of Oncology Bucharest, 252 Fundeni, 022338, Bucharest, Romania; 2 University of Bucharest, Faculty of Chemistry, 4-12 Regina Elisabeta, 030018, Bucharest, Romania; 3 University of Bucharest, Faculty of Physics, MG-11 Magurele, 077125, Bucharest, Romania

**Keywords:** Antioxidant, *Lonicera caerulaea*, experimental tumors, lipid peroxidation, ceruloplasmin, thiol groups

## Abstract

*Lonicera caerulea* is a species of bush native to the Kamchatka Peninsula (Russian Far East) whose berries have been extensively studied due to their potential high antioxidant activity. The aim of our work was to investigate the *in vivo* effects of the antioxidant action of *Lonicera caerulea* berry extracts on the dynamics of experimentally-induced tumors. Our data showed that aqueous *Lonicera caerulaea* extracts reduced the tumor volume when administered continuously during the tumor growth and development stages, but augmented the tumor growth when the administration of extracts started three weeks before tumor grafting. Prolonged administration of *Lonicera caerulaea* berry extracts induced the antioxidant defense mechanism in the tumor tissues, while surprisingly amplifying the peripheral oxidative stress.

## Introduction

Many environmental factors play an important role in the etiology of cancers. When cells are exposed to a carcinogen, the latter can directly or indirectly interact with DNA or with other biomolecules. If the carcinogen is co-oxidized and activated in a metabolic pathway that generates reactive oxygen species, this process can damage the endogenous antioxidant defense mechanisms, leading to chromosomal abnormalities that induce neoplasm transformations. For this reason, one approach to cancer prevention is the suppression of DNA- (or other biomolecule-) targeted attack through the increase of antioxidant capacity. On the other hand, many anticancer therapies (e.g., radiotherapy, anthracycline therapy) are based on the induction of targeted cytotoxicity mediated by free radicals. The drawback of this approach is that radicals with longer lifetimes can migrate from their genesis site, attacking biomolecules and generating irreversible damage.

Antioxidant molecules protect the cells against reactive oxygen species by scavenging them or by promoting their decomposition. Antioxidants are potential chemo-preventive agents against cancer, and a diet high in plant-based foods is often associated with decreased risk of cancer development [1,2,3,4,5]. Cytoprotectants from fruits and vegetables include vitamins, minerals and numerous micro-nutrients. Berries contain high levels of antioxidants, such as polyphenol flavonoids and anthocyanins. There is evidence that berry extracts can modulate certain biomarkers of DNA damage, as well as certain indicators of malignant transformation *in vitro* and *in vivo* [6].

In Romania substantial research is oriented towards the identification of natural compounds that may act as antioxidants, therefore many studies have focused on the characterization of either native or acclimatized plants. *Lonicera caerulaea* L. var. Kamchatka (*Caprifoliaceae*) is a fruit-bearing bush species that originated from the forests that grow on the Kamchatka Peninsula in the Russian Far East. This species has been acclimatized in Romania at the Pitesti-Maracineni Research Institute for Fruit Growing [7]. Analyses of the berry extracts revealed high contents of *β*-carotene, polyphenol flavonoids, anthocyanins and vitamin C [8,9]. In the present work, we have investigated the *in vivo* antioxidant action of *Lonicera caerualea* berry aqueous extracts on the dynamics of experimental tumors induced in rats.

## Results and Discussion

### Effects of Lonicera cerulaea berry extracts on the growth of experimental tumors

Previous investigations on *Lonicera caerulea* berries revealed the presence of anthocyanins and polyphenol flavonoids [8], compounds with antioxidant potential. We obtained aqueous berry extracts that contained substances with antioxidant activity, and we decided to determine whether these extracts would have any influence on the neoplasm transformation process. For this purpose, we supplemented with berry extracts the daily diet of Wistar rats bearing Walker 256 carcinoma. This type of experimental tumor was chosen because it does not induce early metastases [10,11,12]. 

We determined the identity and amount of antioxidants in each aqueous berry extract that we used in this study and found that they consistantly contained ascorbic acid, polyphenols and antocyanins (Table 1), while alkaloids and tannins were completely absent (data not shown). 

**Table 1:** 

Compound*	Amount detected
Ascorbic acid	95 ± 0.82 mg /100 mL extract
Phenolic compounds (as gallic acid equivalent)	1.16 ± 0.05 mg /100 mL extract
Total anthocyanins (as cyanidin equivalents)	8.21 ± 0.32 mg /100 mL extract

* The composition of each extract was determined in duplicate; values represent the mean composition of all extracts used ± standard deviation. Only the extracts that were not significantly different in composition (P < 0.05) were used as dietary supplements.

To determine the eventual chemopreventive action of the extracts upon tumor development, we introduced the berry extracts into the animals’ diet starting either three weeks before tumor grafting (lot EM1) or concomitantly with tumor grafting (lot EM2). 

**Figure 1:**
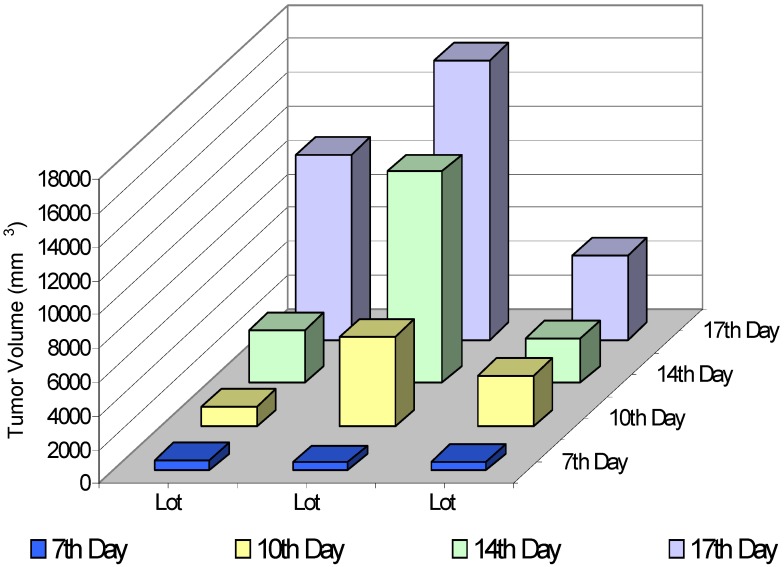
Effect of *Lonicera cerulaea* berry extracts on the tumor growth.

Total berry extracts were administered daily in 1 mL aliquots to carcinoma-bearing Wistar rats. The extracts were administered three weeks before (lot EM1) or at the same time as tumor grafting (lot EM 2). The control group (lot C) consisted of tumor-bearing rats that did not receive berry extracts. The tumor volume was calculated using the formula: V = 0.52 ∙ a ∙ b^2^, where **a** and **b** are the maximum and the minimum tumor diameters, respectively. The data presented are averaged values of tumor volumes corresponding to each lot (10 animals/lot/time point). Time 0 was considered the day when the tumor cells were inoculated.

Figure 1 shows the changes of the tumor volume in both groups of animals treated with berry extracts (EM1 and EM2) and in the tumor-bearing animals that were not given berry extracts (Lot C). The tumor growth was monitored on a daily basis. The tumors were detectable one week after tumor cell inoculation (day 7); henceforth the changes in tumor volumes occurred approximately every three days (i. e., days 10, 14 and 17 from tumor grafting). Surprisingly, the tumor volume was considerably larger when the administration of berry extracts started three weeks before tumor grafting (lot EM1), suggesting that the extracts did not induce a long term protective effect. We were not able to continue the investigation longer because from day 18 the animals belonging to lot EM1 started to die (data not shown). When the administration of berry extracts started concomitantly with tumor grafting (lot EM2), a progressive increase of the tumor volume was noted, but the average tumor volume of these animals was constantly lower than that of the control group. This observation suggested us that in this experimental lot the extracts may have had a protective effect against tumor progression. The ratio between the average tumor volume in the control group (lot C) and the average tumor volume in the treated group (EM2) was 1.17 and 2.21 corresponding to days 14 and 17, respectively. 

### Effect of Lonicera caerulaea berry extracts on the oxidative state of the tumor-bearing animals

To understand why the berry extracts had such different influence on the tumor growth in lots EM1 and EM2, we monitored the biochemical parameters that characterize the oxidative state of the animals. The investigations were done in serum, hepatic and tumor tissues, by testing the following biochemical parameters: lipid peroxidation index (LPI), ceruloplasmin activity (CPA), total thiol groups (TSH) and oxidative stress index (OSI). These parameters were measured on the days we noticed a change in the tumor volumes in any of the lots (i. e., days 7, 10, 14, 17 from tumor grafting).

### Lipid peroxide index (LPI)

**Figure 2:**
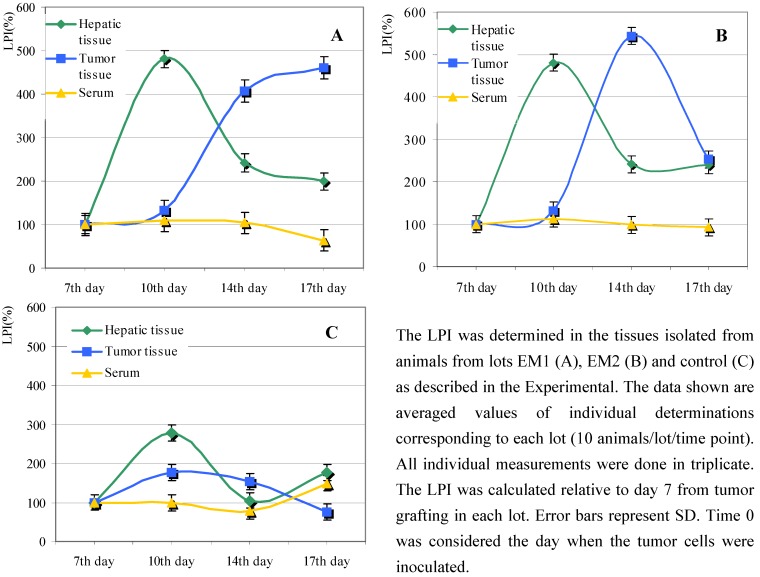
Effect of *Lonicera caerulaea* berry extracts upon lipid peroxid extration index (LPI).

We monitored the levels of lipid peroxides in various tissues isolated from animals, in both experimental models. While there was no significant difference concerning the serum LPI between the berry extract treated lots and the control lot, the level of hepatic LPI was almost twice as high in the case of berry extract fed animals (Figure 2). 

The increase of LPI is a common feature of all tumors due to cell proliferation, and also due to angiogenic transformation of the tumor tissues [13]. We noticed an initial increase of LPI in all tumor tissues isolated from berry extract treated animals (Figure 2A, B). Differences between lots EM1 and EM2 concerning tumor LPI were seen only in the interval between day 14 and day 17 from tumor grafting: thus, after continuously increasing up to day 14, the tumor LPI reached a plateau in lot EM1 (Figure 2A), whereas the tumor LPI for lot EM2 dropped from day 14 to day 17 (Figure 2B). 

The constant increase of LPI in the tumor tissues isolated from lot EM1 (Figure 2A) paralleled the continuously growing tumor average volume (Figure 1). In the case of lot EM2 the tumor exhibited a constant increase of lipid peroxidation until day 14, paralleling the progression of the average tumor volume. In contrast to lot EM1, for lot EM2 we noticed a fall in the levels of LPI from day 14 to day 17, which corresponded to the smaller progression of tumor volume during this period (Figure 1).

### Ceruloplasmin activity (CPA)

**Figure 3:**
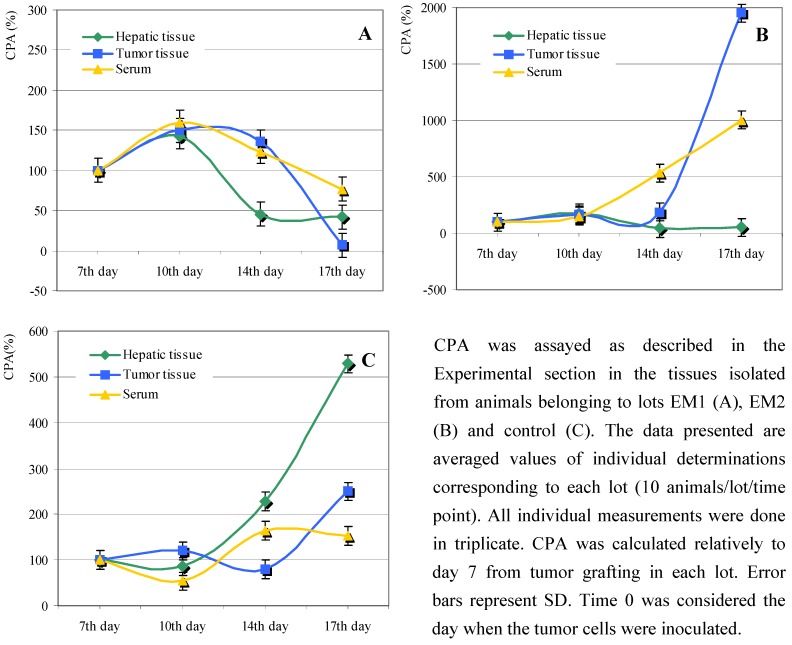
Effect of *Lonicera caerulaea* berry extracts on ceruloplasmin activity (CPA).

Ceruloplasmin is an enzyme with antioxidant activity that acts against lipid peroxidation [14,15]. We therefore decided to monitor the CPA levles in the tissues assayed for LPI. The pattern of the enzymatic activity of ceruloplasmin in all biological samples was similar up to day 10 in lots EM1 and EM2, showing a moderate increase in all tissues investigated (Figure 3A, B). 

Following day 10 from tumor grafting, we noticed significant differences between the EM1 and EM2 lots. Thus, in the case of lot EM1, the enzymatic activity of CPA increased up to day 10 in all monitored tissues and then subsequently decreased more or less abruptly (Figure 3A). Remarkably, the tumor CPA activity decreased dramatically between days 14 and 17 to an almost null value; this could account for the high values of LPI in the same period (Figure 2A). The CPA pattern was different in the case of animals from lot EM2. Thus, CPA in serum increased continuously from day 7 to day 17, indicating a strong response to oxidative stress (Figure 3B). High CP activity was also detected in the tumor tissues, with a notable increase from day 14 to day 17 (more than 10-fold, Figure 3B) which could be correlated with the decrease of LPI in the same period (Figure 2B).

CPA in the serum and in the tumor tissues isolated from lot EM2 increased even more towards the end of the monitoring period. This result was unexpected, since the level of ceruloplasmin in the hepatic tissue decreased considerably towards the end of the experiment (Figure 3B), and the liver is the site of ceruloplasmin biosynthesis. The increased CPA in the serum or tumor tissue may have been caused by an important protein overflow into the bloodstream, probably due to inflammatory process(es) coupled with cellular lysis. 

### Total thiol groups (TSH)

**Figure 4:**
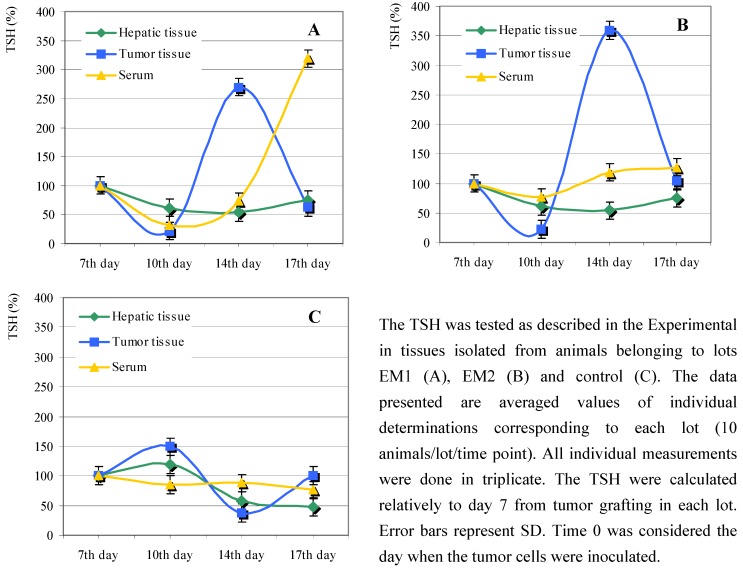
Effect of *Lonicera caerulaea* berry extracts on total thiol groups (TSH).

The animals fed with diets supplemented with *Lonicera caerulaea* berry extracts showed a sinusoidal variation of total thiol groups (TSH) in the tumor tissues. Notably, the maximum was reached around day 14 from tumor grafting, followed by a sharp decrease in the next three days (Figure 4A, B). An increase in the level of the serum TSH was recorded for lot EM1 after day 14 (Figure 4A), that could be correlated with the low CPA in the sera obtained from these animals (Figure 3A). 

To examine what global effect a diet enriched with *Lonicera caerulaea* berry extracts had upon the oxidative state of the tumor-bearing animals, we calculated the oxidative stress index (OSI). The OSI is a non-standardized mathematic parameter, without any biological significance, that can be used to estimate the capacity of the *Lonicera caerulaea* berry extracts to stimulate the antioxidant activity in the organism tested.

**Figure 5:**
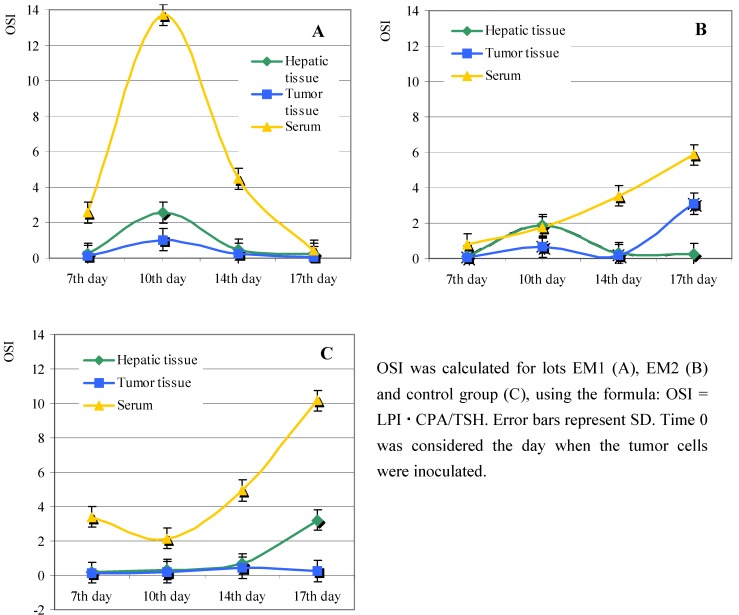
Effect of *Lonicera caerulaea* berry extracts on the oxidative stress index (OSI).

The OSI values were small and presented a similar pattern for the hepatic tissues isolated from animals in lots EM1 and EM2 (Figure 5A, B). Interestingly, there was a sharp increase in the serum OSI calculated for lot EM1, followed by an equally sharp decrease after day 10 from tumor grafting (Figure 5A). By contrast, serum OSI increased after day 10 from tumor grafting in both lot EM2 and in the control group (Figure 5B, C). 

The lowest OSI values were those calculated for the tumor tissues, in both treated lots and the control lot. We noticed however an increase in the OSI calculated for the tumor tissues isolated from lot EM2, starting from day 14 following tumor grafting.

## Conclusions

We have shown here that tumor grafting induced an important oxidative stress that could be alleviated by *Lonicera caerulaea* berry extracts, but only when the extract administration was started concomitantly with the tumor grafting. Continuous administration of the berry extracts starting three weeks before tumor grafting offered only limited protection, having a negative effect on tumor progression. It can be speculated that some of the compounds present in the extracts given in advance, especially the polyphenols, may have augmented the aggressiveness of the cancer cells. In spite of the chemical composition rich in antioxidants, the extracts did not significantly change the overall response to oxidative stress when fed to tumorless animals (data not shown), a response that was however altered in tumor-bearing animals. It is possible that the polyphenols in the extracts given in advance got oxidized and generated minute extra amounts of reactive oxygen species that in the end may have weakened the animals' potential to fight against the cancer cells. In this respect, we found that our extracts constantly contained small amounts of peroxides (an average of 74.1 μmoles peroxides/100 mL extract). These results were similar to those reported by Gruia *et al*., who assessed the modulation effect of various antioxidants on LPI in tumor-bearing mice, when an initial increase of lipid peroxidation was noted, followed by a final decrease, but without reaching the initial values [16].

Prolonged administration of total *Lonicera caerulaea* extracts in Lot EM2 diminished the oxidative stress in the tumor tissues, while amplifying the peripheral oxidative stress and causing no significant changes in the hepatic tissue. It is tempting to believe that constant administration of natural antioxidants from *Lonicera*, *Vaccinum*, *Ribes*, *Sambucus*, etc. can reduce the occurrence of cancer or can alleviate the secondary effects of oncostatic therapy [17,18]. Nevertheless, the contradictory results that we obtained in the two experimental models poses serious questions concerning the beneficial role of natural antioxidants in cancer treatment. We therefore think that both timing and the amount of exogenous antioxidants have to be carefully considered to avoid overproduction of active species during the therapy of cancers.

## Experimental

### Plant material extraction and characterization

*Lonicera caerulaea* L. var. Kamchatka berries grown from the cultivar of the Pitesti-Maracineni Research Institute for Fruit Growing were harvested in May. Berry samples (12 g) were homogenized and boiled with deionised water (20 mL) for 10 minutes, then filtered on sterile cotton wool. The filter cake was extracted twice with boiling water (10 mL). The pooled extract was adjusted to 50 mL with water and was stored at 4°C until used. The total extract contained approximately 0.4 g % dry substance. The quantification of ascorbic acid [19], total anthocyanins [20] and total phenols [21] in the berry extracts was done as described. 

### Animals

Male Wistar albino rats weighing 200-220 g (2 months old) were used. The rats were fed on normal diet, supplemented or not with berry extracts. The animals were housed in standard boxes with standard laboratory diet and water *ad libitum*. The protocol was approved by the institutional animal ethics committee constituted for the purpose. The *in vivo* experimental models were elaborated on legally based decisions about principles of good laboratory practice.

### Tumor implantation

Approximately 106 tumor cells of Walker 256 carcinoma were implanted intramuscularly in the right flank of the Wistar rats. Two weeks after implantation, the tumor grew locally early in the course of diseases without invading the surrounding organs. 

### Experimental design

The rats were divided into groups, each group consisting of 40 animals per lot. Total berry extracts were administered daily in 1 mL aliquots, to both normal and carcinoma-bearing rats. The extracts were administered: 1) three weeks before tumor grafting (lot EM1); 2) at the same time with tumor grafting (group EM 2). The control group (lot C) was formed of tumor-bearing rats that were not administered berry extracts. At the end of the experiments, animals were fasted overnight and killed by cervical decapitation. Blood was collected and serum was separated out [22]. The tumors and the livers were immediately removed and suspended in ice-cold saline. Tests on whole blood, tumor or hepatic tissue were performed in days 7, 10, 14 and 17 following the tumor grafting for all experimental lots (10 animals from every lot for every time point).

### Oxidative stress assessment

The tumor volume was calculated using the formula: V = 0.52 ∙ a ∙ b^2^, where a and b represent the maximum and the minimum tumor diameters, respectively. The lipid peroxidation index (LPI) was determined by the malonylaldehyde assay [23]. The enzymatic activity of ceruloplasmin (CPA) was assayed as described by Ravin [24]. The total thiol groups (TSH) were assayed as described by Schosinski, using Ellman reagent [25,26,27,28]. All biochemical determinations were done in triplicate and are expressed as mean values. The data presented were calculated as percentage relatively to day 7 following tumor grafting. The oxidative stress index (OSI) was calculated using the formula: OSI = LPI ∙ CPA/TSH. 

### Statistical analysis

The levels of variables in the cancer tissues, hepatic tissues and in the sera were compared with the control and were analyzed statistically. The results in the groups under study are given as median and mean ± SD (standard deviation). To compare data of tumor and control tissues, Walloon’s rank test was used. P<0.05 was considered statistically significant.
