# Correction: Gruia, M.-I. *et al*. The Antioxidant Response Induced by *Lonicera caerulaea* Berry Extracts in Animals Bearing Experimental Solid Tumors. *Molecules* 2008, *13*, 1195-1206

**DOI:** 10.3390/molecules14020893

**Published:** 2009-02-24

**Authors:** Maria Iuliana Gruia, Eliza Oprea, Ion Gruia, Valentina Negoita, Ileana Cornelia Farcasanu

**Affiliations:** 1 Institute of Oncology Bucharest, 252 Fundeni, 022338, Bucharest, Romania; 2 University of Bucharest, Faculty of Chemistry, 4-12 Regina Elisabeta, 030018, Bucharest, Romania; 3 University of Bucharest, Faculty of Physics, MG-11 Magurele, 077125, Bucharest, Romania

In the original published version of this paper [1] we omitted to acknowledge that the work was supported by a grant-in-aid. The acknowledgement is hereby published as follows.
